# A Universal Influenza Vaccine Can Lead to Disease Exacerbation or Viral Control Depending on Delivery Strategies

**DOI:** 10.3389/fimmu.2016.00641

**Published:** 2016-12-26

**Authors:** Cindy Bernelin-Cottet, Charlotte Deloizy, Ondrej Stanek, Céline Barc, Edwige Bouguyon, Céline Urien, Olivier Boulesteix, Jérémy Pezant, Charles-Adrien Richard, Mohammed Moudjou, Bruno Da Costa, Luc Jouneau, Christophe Chevalier, Claude Leclerc, Peter Sebo, Nicolas Bertho, Isabelle Schwartz-Cornil

**Affiliations:** ^1^VIM-INRA-Université Paris-Saclay, Jouy-en-Josas, France; ^2^Institute of Microbiology of the Czech Academy of Sciences, v.v.i, Prague, Czech Republic; ^3^INRA, UE1277, Plate-Forme d’Infectiologie Expérimentale, PFIE, Nouzilly, France; ^4^Institut Pasteur, Unité de Régulation Immunitaire et Vaccinologie, Equipe Labellisée Ligue Contre le Cancer, Paris, France; ^5^INSERM U1041, Unité de Régulation Immunitaire et Vaccinologie, Département Immunologie, Paris, France

**Keywords:** dendritic cells, swine, influenza, human, vaccine, routes of administration

## Abstract

The development of influenza A virus (IAV) vaccines, which elicits cross-strain immunity against seasonal and pandemic viruses is a major public health goal. As pigs are susceptible to human, avian, and swine-adapted IAV, they would be key targets of so called universal IAV vaccines, for reducing both the zoonotic risk and the economic burden in the swine industry. They also are relevant preclinical models. However, vaccination with conserved IAV antigens (AGs) in pigs was reported to elicit disease exacerbation. In this study, we assessed whether delivery strategies, i.e., dendritic cell (DC) targeting by the intradermal (ID) or intramuscular (IM) routes, impact on the outcome of the vaccination with three conserved IAV AGs (M2e, NP, and HA2) in pigs. The AGs were addressed to CD11c by non-covalent binding to biotinylated anti-CD11c monoclonal antibody. The CD11c-targeted AGs given by the ID route exacerbated disease. Conversely, CD11c-targeted NP injected by the IM route promoted T cell response compared to non-targeted NP. Furthermore, the conserved IAV AGs injected by the IM route, independently of DC targeting, induced both a reduction of viral shedding and a broader IgG response as compared to the ID route. Our findings highlight in a relevant animal species that the route of vaccine delivery impacts on the protection induced by conserved IAV AGs and on vaccine adverse effects. Finally, our results indicate that HA2 stands as the most promising conserved IAV AG for universal vaccine development.

## Introduction

Influenza A viruses (IAVs) are responsible of high morbidity and mortality in humans and animals worldwide. Due to constant emergence of antigenic drift variants or novel emerging subtypes, influenza vaccines need to be updated annually. Vaccination with protective antigens (AGs) that are conserved across a broad array of viral variants could mitigate the risks of new pandemic outbreaks. Three conserved AGs could constitute, or be part of, the so called universal flu vaccines: the nucleoprotein NP, the M2e viroporin, and the HA2 stalk domain of the IAV hemagglutinin. NP is among the most important targets for host CD8^+^ T cells (1) and it was shown to afford protection against IAV when provided as a protein vaccine (2), a DNA vector (3), or encoded by heterologous viral carriers (4). M2e is naturally poorly immunogenic in the viral particle. However, when presented in an immunogenic form to the immune system, the M2e AG induces protection *via* anti-M2e IgG that engage Fcγ receptors and antibody-dependent cell-mediated cytotoxicity (ADCC) (5, 6). Finally, immunization with the HA2 triggered partial (7) or even full protective immunity (8, 9), also *via* ADCC mechanism. Most of these results with conserved IAV AG were generated in the mouse model, and promising results were also obtained in horses with NP (10) and in ferrets with M2e (6).

Pigs are susceptible to infection with human and avian adapted strains, as well as swine-adapted strains and are supposed to be potential mixing vessels (11). Persons in contact with pigs may become infected with IAV and conversely, they may transmit human IAV to swine. Pigs are proposed to have been involved in the emergence of the pandemic strain of H1N1 influenza virus in 2009, as the progenitors of the viral genes had been circulating in pigs at least for 10 years before transmission to humans (12). Besides its zoonotic and public health impact, IAV is also responsible of high economical losses in the swine industry. Thus, widening the immune protection of pig against IAV with universal vaccines is highly desirable. Pig and human develop similar flu symptoms upon IAV infection and share physiological, anatomical, and immunological properties, which justify pig as a relevant biomedical model to evaluate universal vaccines against IAV. Notably pigs vaccinated by the intradermal (ID) route with DNA vaccines encoding for M2e and NP developed exacerbated disease following challenge (13), raising concerns about the safety of IAV universal vaccines in pigs and, potentially in humans.

Disease exacerbation by vaccines is a major issue encountered not only in the case of vaccination with conserved IAV AGs in pigs, but also observed with split IAV AGs given intranasally in mice with a TLR4 ligand (14) or for other infections, such as the respiratory syncytial virus in human (15). In these instances, as the initial AG presentation process has primed the host for detrimental responses, improving the initial AG presentation step could differently shape the immune response and result in beneficial outcomes. In that respect, AG targeting to dendritic cell (DC) immunoreceptors has been largely documented to influence the type and the magnitude of B and T cell immune responses (16, 17). The molecular DC targeting is usually achieved by endocytic receptor-specific monoclonal antibodies (mAb) that are covalently linked, or complexed, with AGs. In addition to over 100 promising preclinical studies conducted in mice or *in vitro* using human cells (18), few *in vivo* reports suggest that DC targeting could also be beneficial in large mammals such as in non-human primates, cattle, and pigs (19). In addition, the anatomical site where the DC-targeted AG is delivered could also impact on the shaping of the immune response, given the tissue composition in DC and other interfering cell types and the local specificity of the innate response.

In this work, we assessed whether the delivery of the three conserved IAV AG HA2, M2e, and NP by DC targeting in different anatomical sites could shape the immune response toward protective immunity and avoid disease exacerbation in pigs. We targeted the IAV AG to pig CD11c by generating tetramerized AG fusions to streptavidin that formed tight non-covalent complexes with biotinylated anti-pig CD11c, which we called vaccicomplexes (VCs) (20). This DC-strategy has been successfully used to target the ESAT-6 AG to several mouse immunoreceptors including CD11c and induced significant protection against aerosol-delivered *Mycobacterium tuberculosis* (21). We chose CD11c as it stands as a promising receptor for immunotargeting based on *in vivo* mouse studies and *in vitro* human studies, promoting B and especially T cell responses, including CD8^+^ T cell responses (21–24). We showed that CD11c is highly expressed on swine blood and skin DC and that its pattern of expression on myeloid cell subsets is more similar to the human than to the mouse one, thus making pig a good preclinical model for evaluation of the effect of CD11c targeting (25).

We successfully delivered the three conserved IAV AGs in DC-targeted and non-targeted forms by the ID and intramuscular (IM) routes. We found that the site of VC inoculation had a strong impact on the breadth of immune response and on the clinical and viral outcomes upon infection. The CD11c targeting in the ID route led to disease exacerbation and conversely the IM route, independently of CD11c targeting, favored the antibody response extent to all three AGs and the reduction of viral shedding.

## Materials and Methods

### Antibodies and Adjuvant

The anti-porcine CD11c (anti-CD11c, 3A8 clone, IgG1) and an isotype control (ISC) mouse IgG1 (ISC, 3G8 clone, IgG1) have been generated by our laboratory and described in Ref. (25). The anti-M2e mAb (clone 14C2) was from Invitrogen (Carlsbad, CA, USA). Affinity-purified polyclonal rabbit anti-NP IgG was purchased from Thermo-Scientific, Rockford, IL, USA and affinity-purified polyclonal rabbit anti-HA2 IgG from Biorbyt (Cambridge, UK). Anti-MHC class II mouse mAb (anti-SLA-DR, MSA3 clone, IgG2a) was purchased from Washington State University (USA). Alexa 647-conjugated goat anti-rabbit IgG, with minimal cross-reactivity against ruminant human and mouse proteins, was bought from Jackson ImmunoResearch Laboratories (West Grove, USA). Alexa 488-conjugated goat anti-mouse IgG1 and IgG2a, and Alexa 647-conjugated streptavidin (Invitrogen) were bought from Thermo Fischer Scientific. The mouse anti-swine IFNγ capture mAb (clone pIFNγ) and the biotinylated anti-swine IFNγ (clone PAN) were bought from Mabtech (Nacka Strand, Sweden). The horseradish peroxidase (HRP)-conjugated goat anti-pig IgG Fc was from Bethyl Laboratories (Montgomery, AL, USA), the HRP-conjugated goat anti-rabbit IgG and HRP-conjugated goat anti-mouse IgG were from KPL (Gaithersburg, USA). CpG oligo-dinucleotides (ggTGCGTCGACGCAGggggg) with lower case letters for phosphorothioate linkages and upper case letters for phosphodiester linkages previously shown to be efficient in pigs (26) were bought from Sigma.

### Streptavidin-Fused IAV AG, SDS-PAGE, and Western Blot

**Figure 1:**
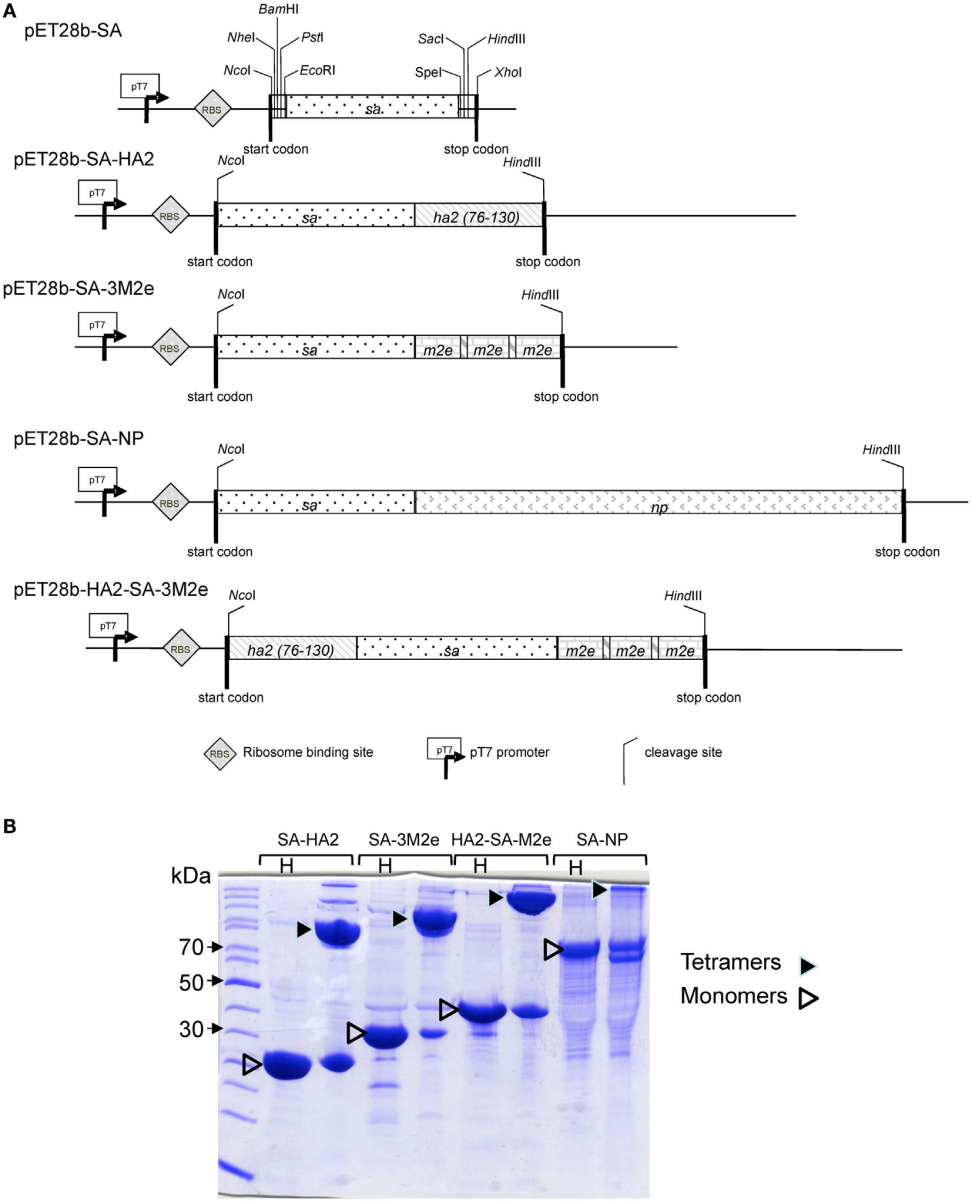
**SDS-PAGE migration of tetrameric SA-HA2, SA-M2e, SA-NP, and HA2-SA-M2a proteins**. **(A)** Schematic depiction of the various pET28b-AG fused to streptavidin (SA–AG) expression constructs. **(B)** SDS-PAGE analysis of the purified soluble and heat-denatured (H) monomers of SA–AG fusion proteins of their native tetramers formed upon dialysis against 50 mM ammonium carbonate pH 8.9. The proteins were separated by Tris–Tricin SDS-PAGE and were visualized by Coomassie blue staining. The tetramers were disrupted by sample heating at 100°C for 5 min (H).

The open reading frames encoding influenza AGs for NP, M2e, and HA2 from IAV sequences of the human A/PARIS/2590/2009 (H1N1) pandemic virus were fused in frame to the 3′ end of a codon-optimized synthetic gene encoding residues 13–139 of streptavidin from *Streptomyces avidinii* (see Figure 1A) (20). The entire coding sequence of the NP gene was PCR-amplified from the cDNA template using specific primers NPfor (5′-*TAGCTAGCACTAGTAGTGACATCGAAGCCATGG-*3′) and NPrev (5′-*TACTCGAGCTAGAATTCACTGTCATACTCCTCTGCAT-*3′). Codon-optimized “Strings™ DNA Fragments” (Thermo Fisher Scientific) synthetic genes were used to clone the genes encoding residues 76–130 of HA2 (RIENLNKKVDDGFLDIWTYNAELLVLLENERTLDYHDSNVKNLYEKVRSQLKNNA), or encoding three consecutive copies of the M2e (3M2e) AG (SLLTEVETPTRSEWESRSSDSSDAAASLLTEVETPTRSEWESRSSDSSDAAASLLTEVETPTRSEWESRSSDSSDAAA) where cysteines 17 and 19 of the original sequence have been replaced by serine residues (underlined), to improve immunogenicity and avoid unwanted disulfide bounds in the chimera, based on previously published works (27, 28). The sequence-confirmed AG fused to streptavidin (SA–AG) fusion proteins were produced in *Escherichia coli* Bl21 λDE3 at 37°C following IPTG induction (0.5 mM). The proteins were extracted by solubilization of washed inclusion bodies with 8 M urea in 50 mM Tris–Cl pH 8 (loading buffer) and the extracts were loaded onto DEAE Sepharose columns. Upon washing of the resin with 10 bed volumes of the loading buffer, the proteins were eluted by a gradient of 2–250 mM NaCl in loading buffer. Fractions containing SA–AG proteins were pooled, diluted fourfold with 50 mM Tris–Cl pH 8, 1 M NaCl (TN buffer), and loaded onto Phenyl Sepharose columns equilibrated in TN buffer. The resin with bound proteins was washed with 10 bed volumes of the TN buffer and the endotoxin contamination was removed by washing with 10 bed volumes of 60% of isopropanol in the TN buffer. Finally, the denatured monomeric SA–AG fusion proteins were eluted with 8 M urea in 50 mM Tris–Cl pH 8. The Phenyl Sepharose and endotoxin removal step was repeated once to reduce the amount of residual endotoxin below <800 EU/mg of SA–AG protein for all AGs. Purified proteins were then dialyzed against 50 mM ammonium carbonate pH 8.9 in order to form soluble and stable tetramers, as verified by SDS-PAGE analysis (Figure 1B). The final tetrameric forms of the SA–AG fusion proteins were stored at 4°C for several days or at −80°C for several months. Purity and long-term stability of the final AGs were controlled using the Tris–Tricin SDS-PAGE gel separation with Coomassie staining (Figure 1B). Protein concentration was determined by the Bradford assay using BSA as calibration standard and tetrameric state of the SA–AG proteins was systematically verified by SDS-PAGE before immunization. The antigenicity of the SA–AG was analyzed by Western blot after under reducing conditions (Figure S1 in Supplementary Material). The M2e–nucleoprotein carrier from the respiratory syncytial virus was used as a positive control (28). After transfer onto nitrocellulose membrane, the blots were blocked in PBS 0.3% Tween 20 and 5% non-fat dry milk, and respectively, reacted with mAb anti-M2e (0.3 µg/ml), rabbit polyclonal IgG anti-NP (0.3 µg/ml), or anti-HA2 (5 µg/ml), followed by washing and decoration with HRP-conjugated anti-mouse IgG (1/10,000) or HRP-conjugated anti-rabbit IgG (1/10,000), respectively. Control blots with irrelevant primary antibodies were performed to control for specificity. Detection was performed using enhanced chemiluminescence kit (ECL, Thermo Fischer).

### Recombinant NP Production

NP gene was cloned in the *E. coli* expression vector pET22b+ (Novagen) between the *Eco*RI and *Xho*I restriction sites, leading to the expression of C-terminal His_6_ fusion protein. The gene coding for NP A/PARIS/2590/2009 (H1N1) was amplified with gene-specific primers (sequences available upon request) using A/PARIS/2590 genetic reverse system plasmid as a template. The sequence of the gene was confirmed by nucleotide sequencing. Competent BL-21 Rosetta cells (Stratagene) transformed with the pET22-NP-HisTag plasmid were cultured to an *A*_600_ of 0.6 in L medium. To express NP, the transformed cells were incubated overnight at 28°C in 1 mM isopropyl 1-thio-β-d-galactopyranoside with agitation. NP purification was purified as previously described (29). Briefly, after centrifugation, the bacterial pellet was resuspended in the lysis buffer, incubated on ice for 1 h, sonicated, and treated with benzonase for 30 min at room temperature. The lysate was clarified by centrifugation for 30 min at 10,000 × *g* at 4°C, and the supernatant was collected and loaded on a Hitrap-IMAC column using the AKTA Purifier-100 FPLC chromatographic system (GE Healthcare). NP-His_6_ was purified by IMAC-Ni^2+^ affinity chromatography followed by size-exclusion chromatography using a Superdex S200 column and Tris 20 mM pH7.4, NaCl 50 mM buffer. After purification, the purified protein were subjected to 12.5% SDS-PAGE analysis and detected by Coomassie brilliant blue staining. The purified NP concentration was determined by the extinction coefficient ε = 57,691.31 M^−1^ cm^−1^ at 280 nm.

### Biotinylated mAb and VCs Formation

Protein G-purified anti-CD11c and ISC mAbs were biotinylated (2:1 ratio) using the EZ-Link Sulfo-NHS-LC kit (Pierce, Rockford, IL, USA). Free biotin was removed by dialysis and the antibody solutions in PBS were sterilized by filtration through 0.2 µm filters. VC were allowed to form on ice 30 min before use, by incubating the biotinylated mAb (biot-anti-CD11c and biot-ISC) with a two times molar quantity of SA–AG.

### Production of the Influenza Challenge Virus

The human-derived A/PARIS/2590/2009 (H1N1) pandemic virus was provided by the National Influenza Center (Northern-France) at the Institut Pasteur, Paris. As previously described, the eight genomic segments of influenza A/PARIS/2590/2009 (H1N1) were amplified with gene-specific primers and cloned into a bidirectional transcription plasmid derived from pRF483 plasmid (30) to generate recombinant viruses. All plasmids’ inserts were verified by nucleotide sequencing. The method used for production of the recombinant A/PARIS/2590/2009 (H1N1) virus was adapted from previously described reverse genetics procedures (31). Briefly, a subconfluent coculture of 293T and MDCK cells in a 35-mm dish was transfected with the eight pRF483 plasmids (0.5 μg of each), using 10 µl of Fugene HD transfection reagent (Roche). After 24 h of incubation at 37°C, the culture medium was removed, and the cells were further incubated for a 48-h period at 37°C in DMEM supplemented with a reduced 2% concentration of fetal calf serum (FCS). The efficiency of reverse genetics was evidenced by a virus-induced cytopathic effect on MDCK cells and further confirmed by titration on MDCK cells. The working stocks of the recombinant viruses were prepared by two successive amplifications in MDCK cells at a multiplicity of infection (MOI) of 10^−3^ for 3 days at 35°C in MEM. Virus titer was determined on MDCK cells using plaque assay procedure as previously described (28). The A/PARIS/2590/2009 (H1N1) viral production and titration was performed in the BSL3 facilities of the VIM-INRA laboratory in Jouy-en-Josas, France.

### Animals and Immunization Experiments

The animal experiments were approved by the French ethical committee #19, i.e., the Comité d’Éthique en Expérimentation Animale Val de Loire, under the number 00783.02 in accordance with national guidelines on animal use. The animal experiments were done at the Plate-Forme d’Infection Expérimentale PFIE-INRA, Nouzilly, France, under the accreditation number for animal experimentation C37-175-3. The immunization and challenge parts were performed under A-BSL1 and A-BSL3 containment, respectively. Large-white pigs were obtained from the INRA conventional breeding unit Unité Expérimentale de Physiologie Animale de l’Orfrasière PAO-INRA, Nouzilly, France.

In the case of experiment 1, pigs (3–4 months of age) were randomly assigned into six groups of six pigs each (half males, half females). Half of the groups received the protein vaccines only, and the other half received the protein vaccines plus CpG (500 µg per injection) at day 0 (d0) and 30 days post first vaccination (dpv), *via* the ID route, in the inguinal area, under general anesthesia (2 mg/kg xylazine and 10 mg/kg ketamine en IM). The protein vaccine groups include the following: (i) one group vaccinated with CD11c-VC-NP (left side) and CD11c-VC-HA2-M2e (right side), (ii) one group vaccinated with ISC-VC-NP (left side) and ISC-VC-HA2-M2e (right side), and (iii) one group with the uncomplexed SA-NP (left side) and HA2-SA-M2e (right side). The VC were preformed for 1 h at 4°C and each pig dose included 50 µg biot-mAb and SA–AG at a 2 M ratio, i.e., 195 µg SA-NP and 100 µg HA2-SA-M2e in a 400 µl volume. The same amounts of uncomplexed SA–AG in a 400 µl volume were used in the corresponding group. Four 100 µl ID spots were injected per vaccine type. Serum samples were collected by jugular venipuncture at d0 and 55 dpv. Pigs were euthanized 55 dpv, and the spleen cells were harvested. In the course of the experiment, one pig died post anesthesia, in the CD11c-VC + CpG group and one in the ISC-VC + CpG group.

In the case of experiment 2, pigs (2 months of age, mix of males and females) were assigned in five groups of eight or nine animals. One group remained non-vaccinated (eight pigs). Two groups received CD11c-VC-NP (left side) and CD11c-VC-HA2 + CD11c-VC-M2e (right side) either ID (eight pigs) or intramuscularly (IM, nine pigs) at d0 and 50 dpv. Two other groups received IS-VC-NP (left side) and ISC-VC-HA2 + ISC-VC-M2e (right side) either ID (eight pigs) or IM (nine pigs). In that case, the VC dose included 50 µg biot-mAb and SA–AG at a 2 M ratio, i.e., 195 µg SA-NP, 85 µg SA-M2e and 81 µg SA-HA2 in a 400 µl volume. Serum was collected at d0 and 75 dpv.

### Viral Challenge

At 75 dpv in experiment 2, pigs were anesthetized and were challenged by the intratracheal route with 6 × 10^6^ PFU pandemic (H1N1) 2009 virus in 5 ml RPMI in the A-BSL3 facilities of the PFIE-INRA facilities in Nouzilly, France. Appropriate equipment was used to protect the personal (ventilated helmets, protective coverall clothing, gloves, and safety shoes). Nasal swabs were collected daily from day 0 to 8 post challenge (dpc) in 500 µl RPMI and immediately frozen at −80°C. Body temperature was monitored by sensor chips placed under superficial skin muscle. Pigs were examined daily and monitored for dyspnea, coughing, nasal discharge, and conjunctivitis. The last two symptoms were the most frequently observed. As there were no obvious differences in symptom intensities across pigs, we established daily clinical scores by the number of observed symptoms over 8 dpc. The global clinical scores correspond to the sum of the daily clinical scores over the 2–8 dpc period.

### Virus Titration

The nasal swab samples were thawed and vortexed for 15 s, centrifuged for 10 min at 600 × *g* and 100 µl of supernatant were titrated by 10-fold dilutions using plaque assays in MDCK cells at 37°C as described (32).

### Ab ELISA

Individual pig sera were assayed for NP, HA2, and M2e-specific IgG by ELISA. Microtiter plates (Immulon 2HB; Thermo LabSystems) were coated overnight at 4°C with 200 ng peptide (M2e, HA2) or 200 ng recombinant NP per well in 100 µl of PBS. Plates were saturated with 5% FCS in PBS—0.05% Tween 20 for 1 h at 37°C. Samples were threefold serially diluted starting at 1:30 and were incubated for 2 h at 37°C. AG-bound antibodies were detected using HRP-conjugated goat anti-pig IgG Fc (Bethyl Laboratories, Montgomery, AL, USA) at 10 ng/ml, incubated for 1 h at 37°C. ULTRA-tetramethylbenzidine substrate (TMB, Thermo-Scientific) was used for the HA2 and M2e ELISA, and regular TMB for the NP ELISA. Absorbance was measured at 450 nm and the results were expressed as endpoint antibody titers, calculated by regression analysis plotting dilution versus *A*_450_. Endpoint titers were calculated as the highest dilution giving twice the absorbance of the negative control.

### Isolation of PBMC, Splenocytes, Migrated Cells from Skin Explants, and Alveolar Macrophages

Pig blood was collected on 1.3 M citrate and mononuclear cells were isolated on Ficoll-Hypaque density gradient (Amersham Bioscience) and used fresh. Spleen cells were isolated by mechanical dissociation followed by filtration on successive 500 µm and 100 µm mesh-sized nylon filters and centrifugation on Ficoll-Hypaque density gradient. Spleen cells were step frozen in FCS + 10% dimethyl sulfoxide (DMSO) and stored in liquid nitrogen. Migrated cells from pig skin explants were obtained from 24 punch biopsies (8 mm), which were floated for 24 h on 10 ml RPMI 1640 medium + 10% FCS and antibiotics. Migrated cells were collected after loading on a 100 µm mesh-sized nylon filter and used fresh. Alveolar macrophages were collected as described, step frozen in FCS + 10% DMSO, and stored in liquid nitrogen (33). Broncho-alveolar lavage (BAL) was performed twice on isolated left lung with 250 ml PBS + 2 mM EDTA and the collected cells were step frozen and stored in liquid nitrogen.

### Flow Cytometry

Pig BAL cells or migrated cells from pig skin biopsies were incubated for 30 min on ice in saturation buffer made of PBS supplemented with 5% horse serum and 5% swine serum. For analysis of biot-anti-CD11c binding on BAL cells, biot-anti-CD11c and biot-ISC were used at 2 µg/ml followed by Alexa 647-conjugated streptavidin. For labeling with VC, VC were first made on ice, and VC corresponding to 1.5 µg/ml biot-mAb + 6 µg/ml SA-NP were added on the cells for 30 min. The cells were then washed and successively incubated with rabbit anti-NP IgG and anti-MHC class 2 (anti-SLA-DR, MSA3 mAb, IgG2a, 2 µg/ml) and followed by highly specific Alexa 647-conjugated goat anti-rabbit IgG (10 µg/ml) and Alexa 488-conjugated goat anti-mouse IgG2a (2 µg/ml). Dead cells were excluded by DAPI labeling. Flow cytometry acquisition was done with a Becton Dickinson Fortessa Flow cytometer and the acquired data were analyzed using FlowJo software (version X.0.6, Tree Star).

### T-Cell Restimulation with Recombinant NP

In experiment 1, spleen cells were thawed from frozen stocks and dead cells were removed on optiprep gradient. In experiment 2, PBMC were used fresh. In both cases, cells were resuspended at a 10 × 10^6^ cells/ml concentration in X-vivo medium (Ozyme, Saint-Quentin-en-Yvelines, France) supplemented with 2% FCS and penicillin/streptomycin and cultured in 96-well plates for 72 h either in medium alone, with 25 µg/ml ConA or with 3 µg/ml recombinant NP.

### IFNγ ELISA

The culture supernatants from the T cell restimulation assay were tested as duplicates in a IFNγ swine ELISA as recommended by the supplier (Mabtech), using 2 µg/ml capture mAb, 0.5 µg/ml biot-anti-IFNγ and 0.5 µg/ml HRP–streptavidin. Regular TMB was obtained in experiment 1 and ULTRA-TMB in experiment 2. The OD values were obtained at 450 nm. The net OD values were calculated as (mean of the OD from duplicated wells + NP) − (mean of the OD from duplicated wells in plain medium).

### Statistical Analyses

Data were analyzed with the GraphPad Prism 5.0 software. Paired *t*-tests were used for comparison of antibody responses between 0 and 55 dpv in experiment 1. A one-way analysis of variance (ANOVA) followed by Dunnett’s post-test was used for comparison of vaccinated groups against control non-vaccinated group. The correlation analysis between viral shedding and antibody response was done bilateral Spearman test. The principal component analysis (PCA) was done with 12 factors, including viral shedding at 3, 4, 5, 6 dpc, global clinical scores, T cell response at 70 dpv, anti-HA2, anti-M2e, anti-NP Ab response on the day of the challenge, and at 5 dpc. Antibody and viral titer data were log transformed. PCA graphics has been produced using FactoMineR R package (http://factominer.free.fr/).

## Results

### Generation of VCs for Targeting Conserved IAV AG to Swine DC

In order to obtain non-covalent complexes of the AGs with the targeting anti-CD11c antibody, we produced SA–AG protein fusions, where the conserved IAV AG, e.g., NP, three repeated copies of the M2e (3M2e) or the HA2 stem segment were fused to the tetramerizing core of SA, as schematically depicted in Figure 1A. Tetrameric forms of purified SA–AG fusion proteins bind biotin-conjugated antibodies to form VC due to the very high affinity of binding interaction between biotin and tetrameric SA (Kd 10^−15^ M). An additional bi-antigenic chimera was next generated by genetically fusing the HA2 AG to the N-terminal and the 3M2e AG to the C-terminal ends of SA, respectively, to yield HA2-SA-3M2e construct, as depicted in Figure 1A. All SA–AG fusions proteins (SA-HA2, SA-M2e, SA-NP, and the HA2-SA-M2e chimera) were purified close to homogeneity by combination of ion exchange and hydrophobic chromatography. As shown in Figure 1B, the dialyzed SA–AG fusion proteins formed stable tetramers that were stable in SDS-PAGE, unless heat-denatured (H) prior to loading. The four SA–AG fusion constructs were recognized by the specific antibodies directed to IAV AGs in Western blot (Figure S1 in Supplementary Material), supporting their expected antigenicity.

**Figure 2:**
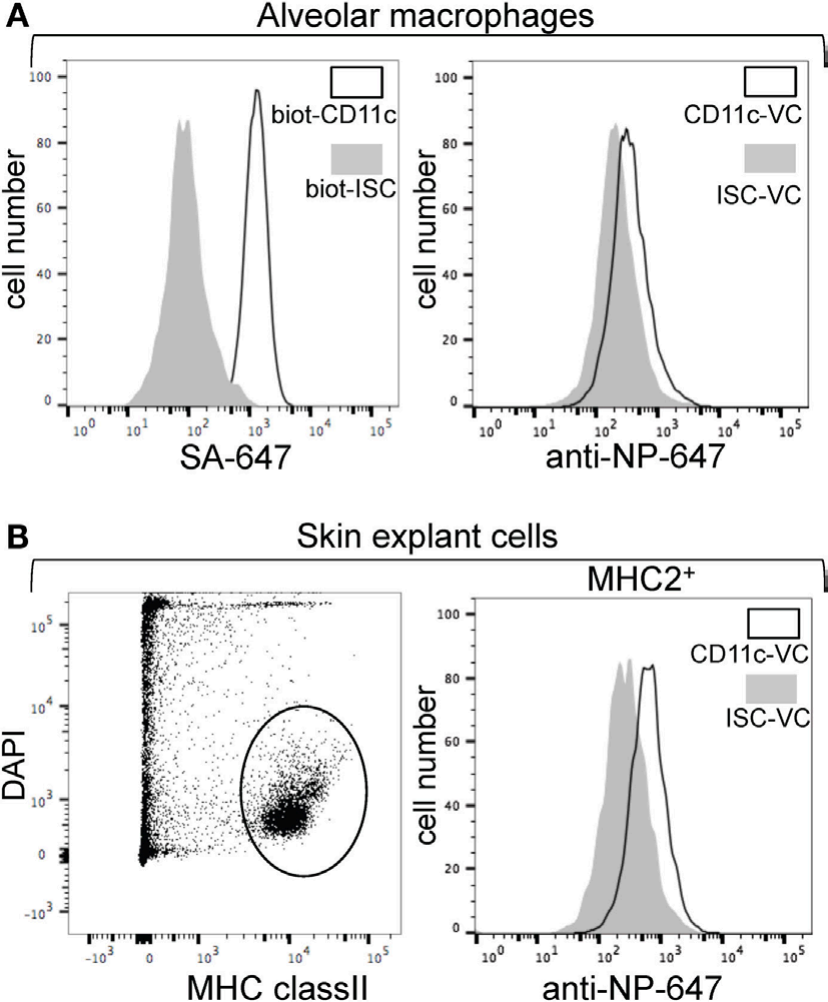
**Binding of CD11c-VC on pig alveolar macrophages and skin dendritic cell (DC)**. **(A)** Binding of CD11c-VC on pig alveolar macrophages. Left panel: broncho-alveolar lavage (BAL) cells were incubated with biot-CD11c monoclonal antibodies (mAb) or biot-isotype control (ISC) followed by Alexa 647-conjugated streptavidin; SSC^high^ FCS^high^ cells (mostly macrophages) were gated and the fluorescent signals were analyzed (biot-CD11c mAb, plain line versus biot-ISC mAb, filled gray). Right panel: BAL cells were incubated with preformed CD11c-VC and ISC-VCn followed by anti-NP rabbit IgG + anti-MHC class II mAb, and Alexa 647-conjugated anti-rabbit + Alexa 488-conjugated anti-mouse IgG2a secondary Ab. After gating the MHC2 class II^+^ cells (alveolar macrophages), the anti-NP fluorescent signals obtained with biot-CD11c (plain line) or biot-ISC mAb (filled gray) are shown. **(B)** Binding of CD11c-VC on pig skin-migrated DC. Migrated cells from skin explants were collected and incubated as in **(A)**. Live (DAPI negative) and MHC class II positive cells were gated and the anti-NP fluorescent signals obtained with biot-CD11c (plain line) or biot-ISC mAb (filled gray) are depicted.

We have previously developed an anti-pig CD11c mAb (3A8 clone) and we showed that it reacts with swine myeloid cells in blood and in skin, and preferentially with conventional dendritic cells (cDC) of the cDC2 type and monocyte/macrophages (25). We reasoned that this mAb is suitable for targeting DC with VC in swine. The anti-CD11c and ISC mAb were biotinylated (CD11c-biot and ISC-biot) and the binding of CD11c-biot on alveolar macrophages was confirmed by Alexa 647-SA (Figure 2A). Preformed CD11c-VC-NP demonstrated a superior binding to both alveolar macrophages or DCs and macrophages from skin as compared to ISC-VC-NP (Figures 2A,B). Thus the VC strategy allows targeting of IAV AG to CD11c on DC in the pig.

### Immunogenicity of VC Delivering Conserved IAV AG upon ID and IM Inoculation

**Figure 3:**
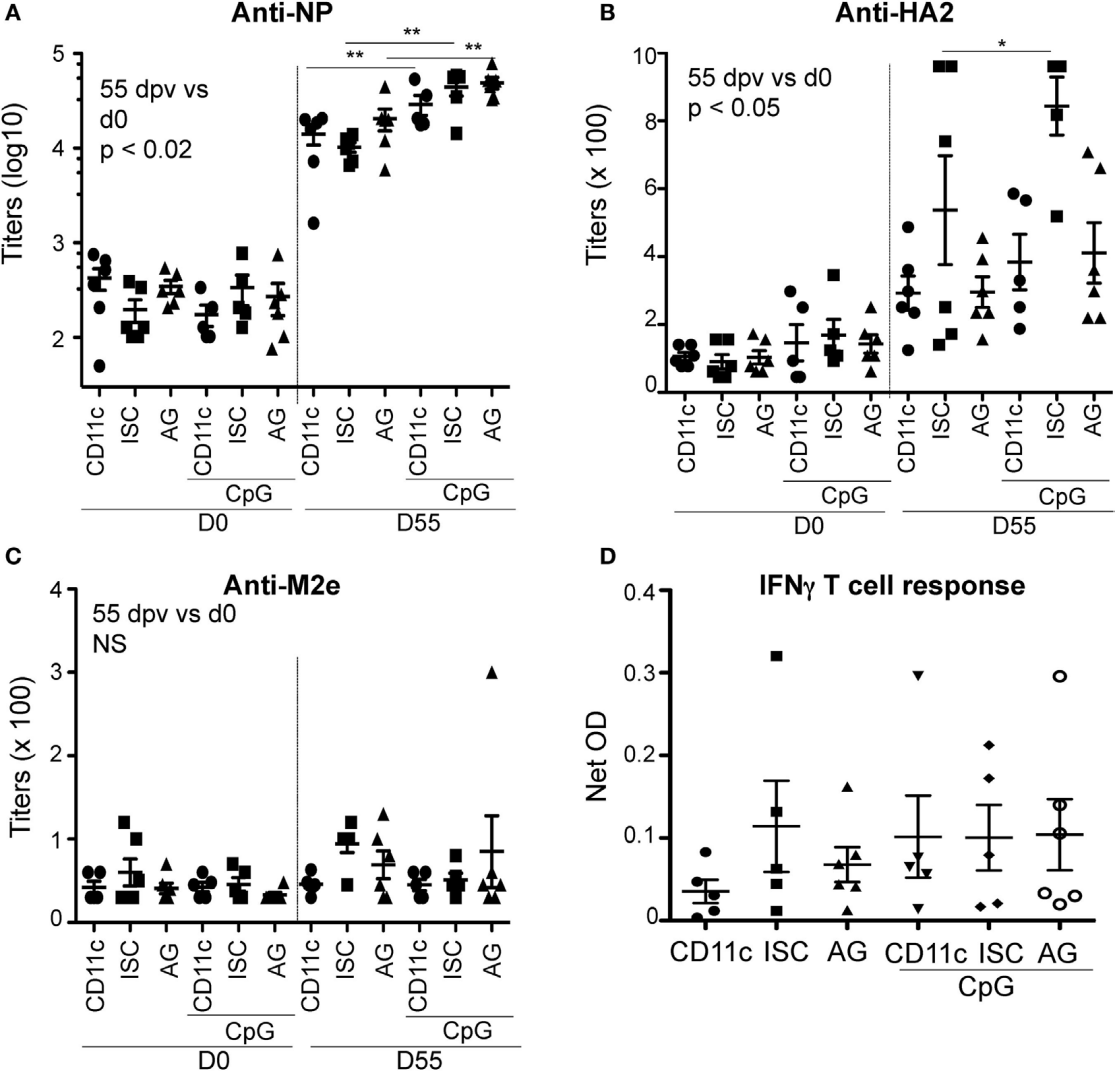
**Ab and T cell responses induced by vaccicomplexes (VCs) upon intradermal (ID) inoculation with and without CpG**. Pigs from experiment 1 were immunized intradermally with CD11c-VC-NP and CD11c-VC-HA2-M2e (designated as CD11c on the figure), isotype control (ISC)-VC-NP and ISC-VC-HA2-M2e (designated as ISC), and uncomplexed AG fused to streptavidin (designated as AG) at 0 and 30 dpv, with or without CpG (500 µg). The anti-NP, HA2, and M2e IgG titers are shown in **(A–C)**, respectively, at 0 and 55 dpv. In **(D)**, the T cell response was assessed by restimulating spleen cells (at 55 dpv) with recombinant NP for 3 days and measuring released IFNγ by ELISA. As recombinant standard was not used, only OD are provided. Each dot corresponds to individual pig values. Means and SEM are shown. Statistical significance between the Ab response at 55 dpv versus d0 was calculated with paired bilateral *t*-tests and significance was always found in the case of anti-NP (*p* < 0.02), and anti-HA2 IgG (*p* < 0.05). Comparison between vaccinated groups at 55 dpv was done with a one-way ANOVA and a Newman–Keuls multiple comparison test, and significance is shown (**p < 0.01, *p < 0.05).

As the dermis is rich in CD11c^+^ DC capable to bind targeted VC, we first decided to inject the CD11c-VC, ISC-VC, and the uncomplexed SA–AG intradermally in pigs (experiment 1). The SA–AG were SA-NP and the HA2-SA-M2e chimera (see Materials and Methods). We also tested the benefit of adding an adjuvant to activate the targeted DC. Mineral or organic adjuvants may interfere with DC targeting, as we observed with the squalene-type of adjuvant (data not shown). Thus addition of a TLR ligand was evaluated. To the best of our knowledge, the only TLR ligands showing *in vivo* adjuvant properties in pigs are CpG oligo-dinucleotides (26). We thus added 500 µg CpG to VC and uncomplexed SA–AG in half of the immunized pigs. The animals were from a conventional breeding unit and thus had significant levels of preexisting Ab against NP and HA2 at the time of vaccination, in line with the fact that a large fraction of the pig population is immune to influenza (34) (Figures 3A–C). All vaccine regimens significantly increased the anti-NP and anti-HA2 IgG levels measured at 55 dpv relatively to d0 (*p* < 0.02 and 0.05, respectively, paired bilateral *t*-test), but not the anti-M2e levels (Figures 3A–C). CpG slightly but significantly improved the anti-NP IgG response and had marginal effect on the anti-HA2 IgG response (Figures 3A–C). CD11c-VC, ISC-VC, and the uncomplexed SA–AG yielded similar efficacy on the antibody response, whether CpG was included or not. There was no differences on the IFNγ response of splenic T cells to NP restimulation at 55 dpv between the different vaccinated groups (Figure 3D). There was no correlation between the preexisting anti-NP and anti-HA2 IgG levels and the measured B and T cell responses to VC and uncomplexed SA–AG, suggesting that preexisting Ab did not interfere on these responses (Figure S2 in Supplementary Material). Thus in our conditions, VC and uncomplexed SA–AG stimulated B and T cell response against NP and HA2 in pigs; however, CD11c targeting did not improve immunogenicity upon ID inoculation.

**Figure 4:**
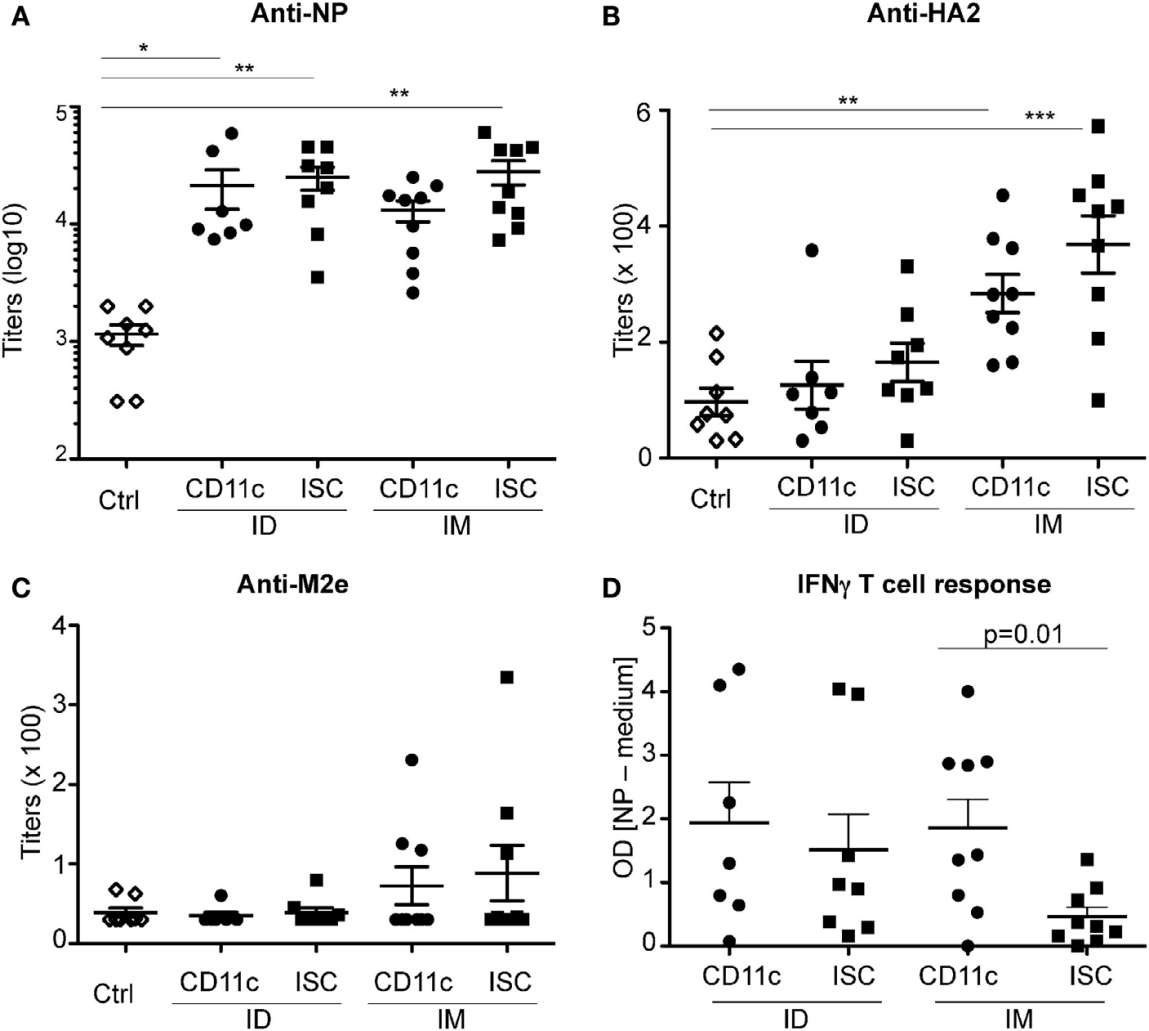
**Ab and T cell responses induced by vaccicomplexes (VCs) upon intradermal (ID) and intramuscular (IM) inoculation without adjuvant**. Pigs from experiment 2 were immunized either by ID or IM routes, with CD11c-VC-NP + CD11c-VC-HA2 + CD11c-VC-M2e (designated as CD11c), or isotype control (ISC)-VC-NP + ISC-VC-HA2 + ISC-VC-M2e (designated as ISC) at 0 and 50 dpv. The anti-NP, HA2, and M2e IgG titers are shown in **(A–C)**, respectively, at 0 and 75 dpv. In **(D)**, the T cell response was assessed by restimulating fresh PBMCs collected at 70 dpv with recombinant NP for 3 days and measuring released IFNγ by ELISA. As recombinant standard was not used, only DO are provided. Each dot corresponds to individual pig values. Means and SEM are shown. Comparison between vaccinated groups and the control non-vaccinated group was done with a one-way ANOVA and a Dunnett’s comparison test, and significance is shown (***p < 0.001, **p < 0.01, *p < 0.05).

Since untargeted VC and SA–AG injected in the dermis may already be efficiently captured by the rich network of local DC, the additional targeting to CD11c does not necessarily provide any advantage at the capture step. We thus compared the immunogenicity of VC injected by ID and IM routes in experiment 2, at the end of which pigs were challenged with pandemic (H1N1) 2009 virus. Given the very high cost and logistics issues of such type of large animal experiments, the comparisons of every potential immunogen and routes of immunization were not feasible and we had to select a limited set of vaccine regimens. Therefore, we decided to compare the immunogenicity of CD11c-VC and ISC-VC delivered by the IM and ID routes without the use of adjuvant, because (i) avoiding adjuvant is seen as one of interesting aspects of the DC-targeting approach and (ii) adjuvant can mask the DC-targeting effect (35). Furthermore CpG showed only minor effect on immune response in experiment 1. We also excluded uncomplexed SA–AG which did not show any immunogenicity difference with VC in experiment 1. As we did not observe Ab responses to M2e in experiment 1, where the HA2-SA-M2e chimera were used, we chose to generate VC where SA-HA2 and SA-M2e were separated, in case that the M2e-antigenic motives had been buried in the HA2-SA-M2e chimera. In this experiment, we also included a group of unvaccinated pigs (control) and we analyzed the Ab response to VC by comparison with this group. The vaccinated pigs presented higher levels of anti-NP IgG at 75 dpv compared to control pigs (Figure 4A). Only pigs injected with VC *via* the IM route presented significant levels of anti-HA2 IgG compared to control pigs (Figure 4B); in addition, three pigs in the CD11-VC and ISC-VC groups injected by the IM route developed anti-M2e titers above 100 (Figure 4C), which had not been obtained by the ID administration of either experiment 1 and 2. The IFNγ response of fresh PBMC collected at 70 dpv to NP was not statistically different between the CD11c-VC and the ISC-VC group vaccinated by the ID route (Figure 4D) but it was significantly higher in the CD11c-VC versus ISC-VC group vaccinated by the IM route (*p* = 0.01, Figure 4D). Due to time and effort constraints, we have not measured the T cell response in the control pigs. There again no correlation was found between preexisting levels of anti-HA2 and NP IgG and the measured immune responses (data not shown). Thus the IM route triggered a broader response to the conserved IAV AG than the ID route, and presented an advantage to the CD11c-targeted VC for promoting T cell response which was not observed in the ID route.

### Induction of Partial Viral Protection with VC Inoculated IM and Induction of Disease Exacerbation with VC Inoculated ID

**Figure 5:**
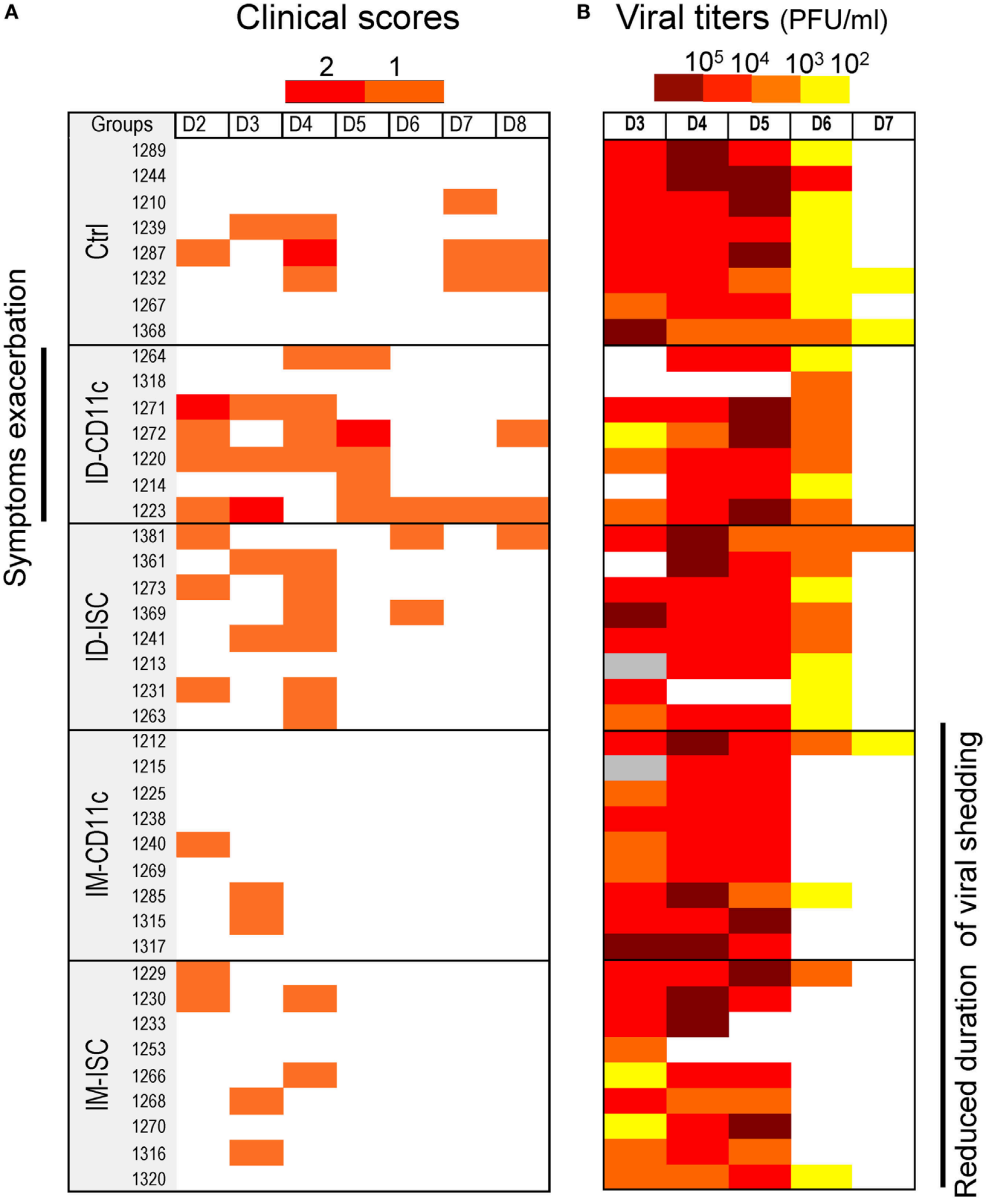
**Clinical symptoms (A) and viral detection (B) in nasal swabs of pigs immunized with vaccicomplexes**. Pigs from experiment 2 (see Figure 4) were challenged by the intratracheal route with pandemic (H1N1) 2009. **(A)** Clinical symptoms, i.e., nasal discharge or conjunctivitis, monitored for each pig from 2 to 8 dpc are reported with a color code, with one symptom in orange, two symptoms in red. Pigs are designated by their breeding number and presented in their assigned groups under the gray tag. **(B)** Viral detection in nasal swab was done daily for each pig from 3 to 8 dpc using plaque assay. PFU/ml superiors to 10^5^, 10^4^, 10^3^, and 10^2^ are represented by a color code from dark red to yellow, respectively. A white rectangle corresponds to absence of detected virus.

Pigs from experiment 2 were challenged with pandemic (H1N1) 2009 by the intratracheal route at 75 dpv. All pigs showed a temperature peak at 1 dpc without differences between groups (data not shown). Symptoms were mild in the control group and were expressed as nasal or oral discharges and conjunctivitis (Figure 5), in agreement with other studies (36). Whereas vaccinated pigs by the IM route showed a tendency to express less symptoms with a shorter duration than the controls, the groups vaccinated intradermally with CD11c-VC, presented disease exacerbation (Figure 5; Figure S3 in Supplementary Material). Virus was detected in nasal swabs in all pigs between 3 and 5 dpc and was not detected at day 8 (Figure 5). Interestingly, most pigs vaccinated by the IM route did not shed virus at 6 dpc whereas the pigs in the control and in the ID vaccinated group still did (Figure 5). These results indicate that the immune response triggered by VC injected *via* the IM route affects viral shedding duration, whereas the immune response triggered by CD11c-VC injected *via* the ID route is detrimental on clinical symptoms upon challenge.

### Reduction of Viral Shedding Correlates with Anti-HA2 and Anti-M2e IgG Responses and not with anti-NP Responses Induced by VC

**Figure 6:**
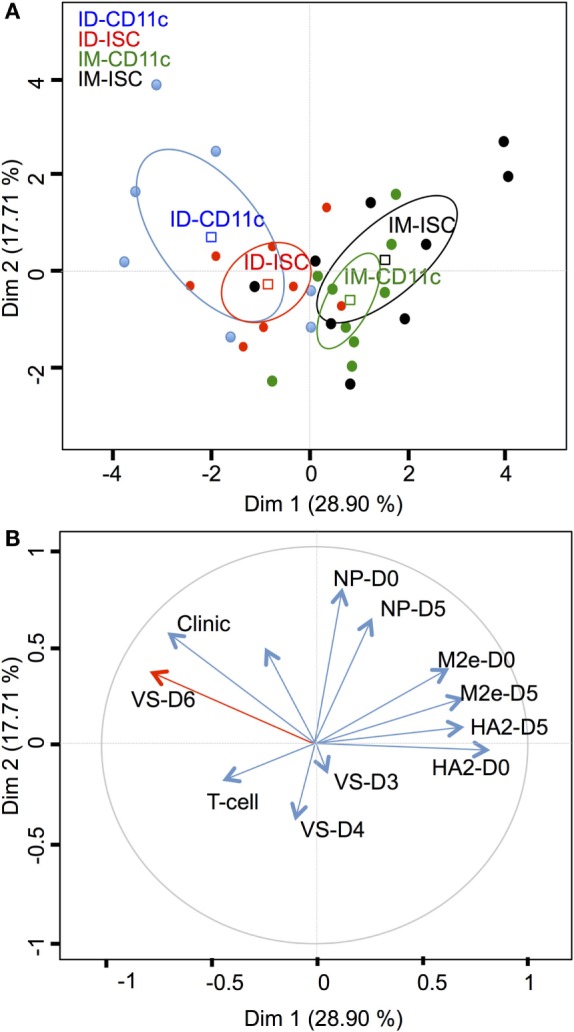
**Principal component analysis (PCA) analysis of the viral shedding and immune responses of vaccicomplexes (VCs)-vaccinated pigs**. **(A)** PCA plot of the responses of pigs is depicted with each pig represented as a dot in a specific color according to its group assignment: CD11c-VC intradermal (ID-CD11c, blue), isotype control (ISC)-VC intradermal (ID-ISC, red), CD11c-VC intramuscular (IM-CD11c, green), ISC-VC intramuscular (IM-ISC, black). PC1 explained 28.9% of the total variation between pigs and PC2 explained a further 17.71% of the variation. **(B)** PCA loading for each individual input variable: viral shedding at 3, 4, 5, 6 dpc (VS-D3, VS-D4, VS-D5, VS-D6), global clinical symptoms over 2–8 dpv (Clinic), T cell response at 70 dpv (T-cell), anti-HA2, anti-M2e, anti-NP Ab response on the day of the challenge (HA2-D0, M2e-D0, NP-D0) and at 5 dpc (HA2-D5, M2e-D5, NP-D5). Note that the day of challenge (D0 in Figure 6) corresponds to 75 dpv in Figure 4.

We used PCA to identify the result variables that were responsible for the variation of the data across vaccinated pigs. The variables included viral shedding at 3–6 dpc, global clinical scores, IgG titers at 75 dpv and 5 dpc, and T cell response at 70 dpv. As depicted in Figure 6A, the route of inoculation (PC1) contributes to 28.9% of the variation between pigs. Figure 6B shows the PCA loading for each variable and indicates that viral shedding at 6 dpc opposes to anti-HA2 and anti-M2e IgG levels at the day of challenge and 5 dpc with anti-HA2 at the day of challenge having the highest weight. A two-tailed Spearman test between the viral shedding at 6 dpc and anti-HA2, anti-M2e, and anti-NP at 75 dpv and 5 dpc confirmed that viral shedding at 6 dpc was anti-correlated mainly with anti-HA2 (*r* = −0.7, *p* < 0.001 at 75 dpv and *r* = −0.5 at 5 dpc, *p* < 0.01) and less so with anti-M2e (*r* = −0.37 at 75 dpv and −0.33 at 5 dpc, *p* < 0.05) and did not anti-correlate with anti-NP IgG response (Figure S4 in Supplementary Material). Overall our data analysis confirms that the route of VC inoculation has a dominant impact on the outcome of the immune response, and indicates that HA2 stalk stands as the most promising conserved IAV AG for the design of universal vaccine.

## Discussion

Our results reveal that vaccination with conserved IAV AGs can induce disease exacerbation in the pig model, supporting the initial observation obtained with NP and M2e-based DNA vaccines (13). Whereas we aimed at preventing this detrimental effect with DC targeting, on the contrary we found that CD11c-targeting promoted disease exacerbation when injected ID. Conversely partial viral protection was obtained when the IAV AGs were delivered by the IM and not the ID route, independently of DC targeting. Despite that higher T cell response was obtained with CD11c targeting in the IM route, it did not translate in better protection. Finally, viral clearance correlated with anti-HA2 and M2e IgG levels and not with anti-NP IgG levels.

The IM route revealed to be more suitable than the ID route to induce the desired immunity in our setting and to observe a benefit of CD11c targeting on the T cell response. To our knowledge, previous reports on DC targeting of protein vaccines utilized mainly the intravenous route, less often the subcutaneous, the intranasal and the IM routes, and rarely the ID route (19). We speculated that the high bolus of AG in the small and confined ID compartment would favor the capture by the local DC, thus not offering advantage to CD11c targeting. Furthermore, the ID route is reactogenic and triggers rapid local inflammation, which could result in rapid AG degradation by the recruited myeloid cells (neutrophils, monocytes) that could explain the lack of response to VC-HA2 and VC-M2e injected ID in experiment 2. Furthermore, the reactogenicity of the ID site may provide a priming milieu for subsequent detrimental response upon IAV exposure. Indeed, CRX-601 TLR4-L combined with split-influenza AG administered by the intranasal routes and not by the subcutaneous route, induced disease exacerbation after challenge with IAV (14). Expansion of vaccine-primed Th17 cells was responsible of the increased morbidity. We indeed observed a local inflammation after VC and uncomplexed SA–AG upon ID administration, which disappeared after 4–5 days. The endotoxin levels per injection were below 150 EU, which is below the accepted endotoxin limit in humans (37); nevertheless, endotoxins may have contributed to the local response and possibly to the deleterious priming.

We chose to target DC with an in-house developed mAb to swine CD11c as previous successful CD11c targeting was achieved *in vivo* in mice (23, 24, 38, 39) and *in vitro* in humans (40). In addition, the pattern of CD11c expression on pig skin and blood myeloid cell subsets whose organization aligns across species (41–43), is closer to the human than to the mouse one, making pig a pertinent preclinical model to evaluate this type of targeting. Indeed, CD11c shows a higher expression on cDC2 than on cDC1 and is highly expressed on skin monocyte-derived cells in the pig and human (25, 44) and not in the mouse (45). Consequently, when injected in pigs, VC were not uniquely targeted to cDC, which are the most potent AG-presenting cells and could have even been targeted to counterproductive cells, for instance monocyte-derived cells or even granulocytes. The variation in targeting selectivity across species might explain outcome differences of DC-targeting strategies between mice and large mammals (19). Thus more selective immunoreceptors could reveal to be better targets, such as XCR1 which is specifically expressed on cDC1 in all mammalian species investigated so far and for which we recently validated a suitable targeting tool (25). Mouse and human studies demonstrated that CLEC9A, which is expressed on cDC1 and plasmacytoid DC (pDC) (46), and CLEC10A, which is expressed on cDC2 and monocyte-derived DC (17), are promising molecular targets for DC targeting. However, pig do not seem to possess a functional CLEC9A gene and, despite the existence of a putative swine CLEC10A coding sequence, we were unable to identify a corresponding mRNA nor to show any specific interaction of pig skin DC with Tn-glycopeptide, the ligand of CLEC10A in other mammals (unpublished data). Indeed, the selection of the immunoreceptor is key for the success of immunotargeting, but despite intensive research, the requested criteria for improved responses have not yet been identified (47). It is assumed that both the DC subtype and/or the endosomal compartment of AG routing consecutive to targeting are important parameters as receptor expression level, proportion of surface turnover, and speed of receptor internalization do not impact on AG presentation efficiency (47).

The VC strategy with SA–AG and biot-mAb has the great advantage of being simple and versatile (21). However, this approach presents potential caveats. First, the biotinylation and the large tetramerised AGs may interfere with the interaction between the Ab and its targeted immunoreceptor. The used mouse IgG1 could be intrinsically immunogenic or lead to immune competition with the vaccine AG as well as interact with the pig Fc receptors. We measured anti-mouse IgG-pig IgG by ELISA in the pig serum of the experiment 1 and found low titers (200 ± 186 in all vaccinated pigs, without difference across groups). Finally, it should also be stressed that the influenza AG delivered by our VCs are most likely not in their native conformation. The native M2 is tetrameric while native HA2 is trimeric in the viral particles (48, 49). However, several reports, including work from our research groups, have shown that non-tetrameric M2e and non-trimeric HA2 AGs can also elicit antibodies that are protective against a viral challenge (8, 27, 28, 50). Importantly, the mechanism of protection induced by such non-native M2e and HA2 structures was shown to involve ADCC (9, 51) and not the classical neutralization by antibodies. The conformation required to elicit antibodies against HA2 and M2e that are efficient in ADCC has not been determined to our knowledge. Nevertheless, it remains very possible that the non-native conformation of the influenza AG in the VCs accounts for the limited protective immunity achieved here.

Many preclinical and clinical studies concluded on the requirement to activate DC together with immunotargeting in order to achieve optimal response. CD40L and TLR ligands have been largely used for that purpose, including CpG in mouse models (16). However in the mouse, both pDC and cDC respond to TLR9 stimulation whereas in human, only pDC express TLR9 (52). A very recent study in pigs show that although cDC and monocyte-derived DC express TLR9 mRNA, they do not upregulate cytokine nor costimulatory molecule expression upon exposure to CpG, unless cocultured with pDC (53, 54). As the skin does not contain pDC at steady state (55), it may explain the low adjuvant effect of CpG given by the ID route. We decided to perform experiment 2 without adjuvant, because (i) avoiding adjuvants would be a major advantage in clinical applications of DC targeting, (ii) there are several examples of improved immune responses to DC-targeted AGs delivered in the absence of adjuvants (18, 56) and specifically in the case of CD11c (22, 57), and (iii) adjuvant did masked the effect of DC targeting in macaques (35). However, adjuvants could be beneficial to promote the B and T cell responses induced by VC in the IM route. Furthermore the reduction of viral shedding—certainly limited in this study—correlated with the anti-HA2 and M2e IgG levels induced by VC, encouraging the use of these AGs for universal IAV vaccine development in the pig as a pertinent animal model. We think that the anti-HA2 and anti-M2e Ab levels could be much further increased and achieve better viral protection, if VC would be combined with suitable adjuvants. Strong efforts should be pursued to identify non-mineral/non-organic adjuvants and their formulation for the pigs.

Preclinical development of universal IAV vaccines have been largely assessed using influenza-naive small animal models. However, vaccination strategies are influenced by preexisting influenza-specific immunity (58, 59). In the present study with conventional pigs, the animals presented preexisting levels of anti-NP and HA2 Ab, as it is very often the case in natural field condition. These Ab can either result from passive immunity with maternal transfer or active immunity in case of exposure to the virus, which is not easy to analyze *a posteriori*. In any event, we did not observe a negative correlation between the levels of preexisting Ab and the vaccine-induced Ab or T cell responses, indicating that immunization with conserved IAV AG in VC did not appear to be affected by preexisting Ab.

Our findings have important implication in the vaccinology field. Indeed, we showed that the ID route, despite being rich in DC, was not suitable to induce the desired immunity with our DC-targeted strategy. Worst, the ID route led to disease exacerbation. Conversely in contrast with the ID route, the IM route permitted to (i) unravel the benefit of DC targeting on the T cell response, (ii) generate a broader IgG response against the three conserved AG, and (iii) confer partial protection. Thus, our results also emphasize that the route of inoculation is of major importance to assess molecular DC targeting and that the IM route should be especially considered. And on the applied side, the IM route also offers the advantage to be classically used, easy to perform, and less painful than the ID route.

## Author Contributions

CB-C, CD, OS, EB, CU, C-AR, MM, BC, CC, NB, and IS-C performed laboratory experiments (immunoassays, virology, VC construction, and production). LJ performed the PCA analysis. CB, OB, and JP performed animal experiments. CL, PS, CC, NB, and IS-C designed experiments, directed research, and revised the manuscript. IS-C prepared figures and wrote the manuscript.

## Conflict of Interest Statement

CL, PS and OS are inventors of the issued patent “A versatile delivery system for antigens or biologically active molecules”. EP 09290987.8-1222, 21.12.09. No licence nor royalties. The other authors declare no conflict of interest.

## Abbreviations

IAV, influenza A virus; ADCC, antibody-dependent cell-mediated cytotoxicity; AG, antigen; SA–AG, antigen fused to streptavidin; APC, antigen-presenting cell; BAL, broncho-alveolar lavage; biot-mAb, biotinylated mAb; cDC, conventional DCs; d0, day 0; dpc, day post challenge; dpv, day post vaccination; DC, dendritic cell; DMSO, dimethyl sulfoxide; FCS, fetal calf serum; HRP, horseradish peroxidase; ISC, isotype control; ID, intradermal; IM, intramuscular; mAb, monoclonal antibodies; pDC, plasmacytoid DC; VCs, vaccicomplexes; TMB, tetramethylbenzidine.
