# Corrigendum: A Universal Influenza Vaccine Can Lead to Disease Exacerbation or Viral Control Depending on Delivery Strategies

**DOI:** 10.3389/fimmu.2017.00831

**Published:** 2017-07-18

**Authors:** Cindy Bernelin-Cottet, Charlotte Deloizy, Ondrej Stanek, Céline Barc, Edwige Bouguyon, Céline Urien, Olivier Boulesteix, Jérémy Pezant, Charles-Adrien Richard, Mohammed Moudjou, Bruno Da Costa, Luc Jouneau, Christophe Chevalier, Claude Leclerc, Peter Sebo, Nicolas Bertho, Isabelle Schwartz-Cornil

**Affiliations:** ^1^VIM-INRA-Université Paris-Saclay, Jouy-en-Josas, France; ^2^Institute of Microbiology of the Czech Academy of Sciences, v.v.i, Prague, Czech Republic; ^3^INRA, UE1277, Plate-Forme d’Infectiologie Expérimentale PFIE, Nouzilly, France; ^4^Institut Pasteur, Unité de Régulation Immunitaire et Vaccinologie, Equipe Labellisée Ligue Contre le Cancer, Paris, France; ^5^INSERM U1041, Unité de Régulation Immunitaire et Vaccinologie, Département Immunologie, Paris, France

**Keywords:** dendritic cells, swine, human, vaccines, routes of administration

In the original article, there was an error in the CpG oligo-dinucleotide sequence used as adjuvant.

A correction has been made to the section “Materials and Methods”, paragraph “Antibodies and adjuvants”: CpG oligo-dinucleotides 5′-ggTGCATCGATTTATCGATTATCGATGCAGggggg-3′ with lower case letters for phosphorothioate linkages and upper case letters for phosphodiester linkages previously shown to be efficient in pigs (26) were bought from Sigma.

The authors apologize for this error and state that this does not change the scientific conclusions of the article in any way.

## Conflict of Interest Statement

CL, PS, and OS are inventors of the issued patent “A versatile delivery system for antigens or biologically active molecules.” EP 09290987.8-1222, 21.12.09. No license nor royalties. The other authors declare no conflict of interest.

