# Supplementary material for: A Universal Influenza Vaccine Can Lead to Disease Exacerbation or Viral Control Depending on Delivery Strategies
Source: Front Immunol. 2016 Dec 26;7:641. doi: 10.3389/fimmu.2016.00641 (PMC5183740; doi:10.3389/fimmu.2016.00641)
Supplement: Supplementary file 1 [file Presentation_1.PPTX]

## Slide 1
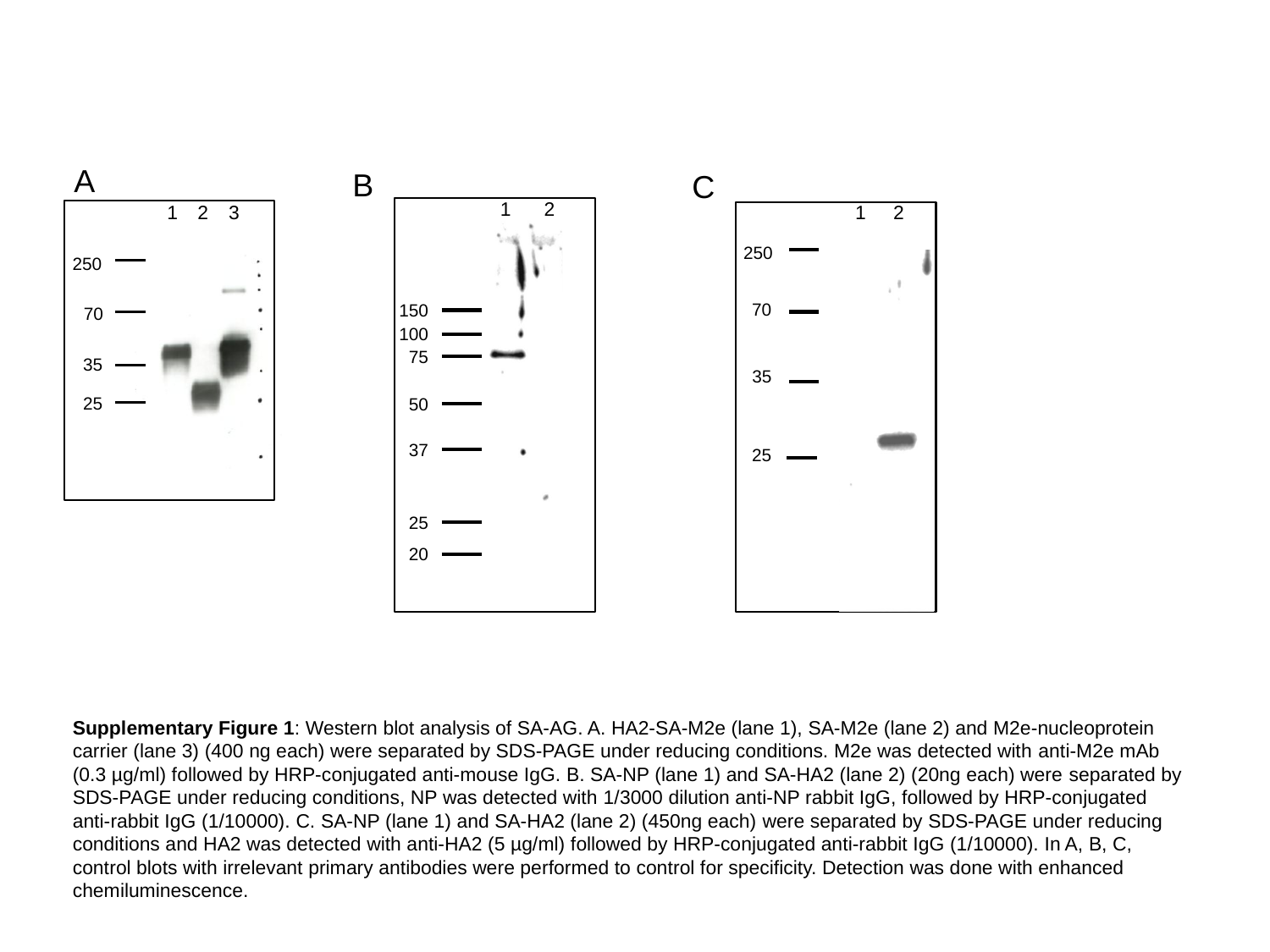

A
B
C
1
2
2
3
1
1
2
250
250
70
150
70
100
75
35
35
25
50
37
25
25
20
Supplementary Figure 1: Western blot analysis of SA-AG. A. HA2-SA-M2e (lane 1), SA-M2e (lane 2) and M2e-nucleoprotein carrier (lane 3) (400 ng each) were separated by SDS-PAGE under reducing conditions. M2e was detected with anti-M2e mAb (0.3 µg/ml) followed by HRP-conjugated anti-mouse IgG. B. SA-NP (lane 1) and SA-HA2 (lane 2) (20ng each) were separated by SDS-PAGE under reducing conditions, NP was detected with 1/3000 dilution anti-NP rabbit IgG, followed by HRP-conjugated anti-rabbit IgG (1/10000). C. SA-NP (lane 1) and SA-HA2 (lane 2) (450ng each) were separated by SDS-PAGE under reducing conditions and HA2 was detected with anti-HA2 (5 µg/ml) followed by HRP-conjugated anti-rabbit IgG (1/10000). In A, B, C, control blots with irrelevant primary antibodies were performed to control for specificity. Detection was done with enhanced chemiluminescence.

## Slide 2
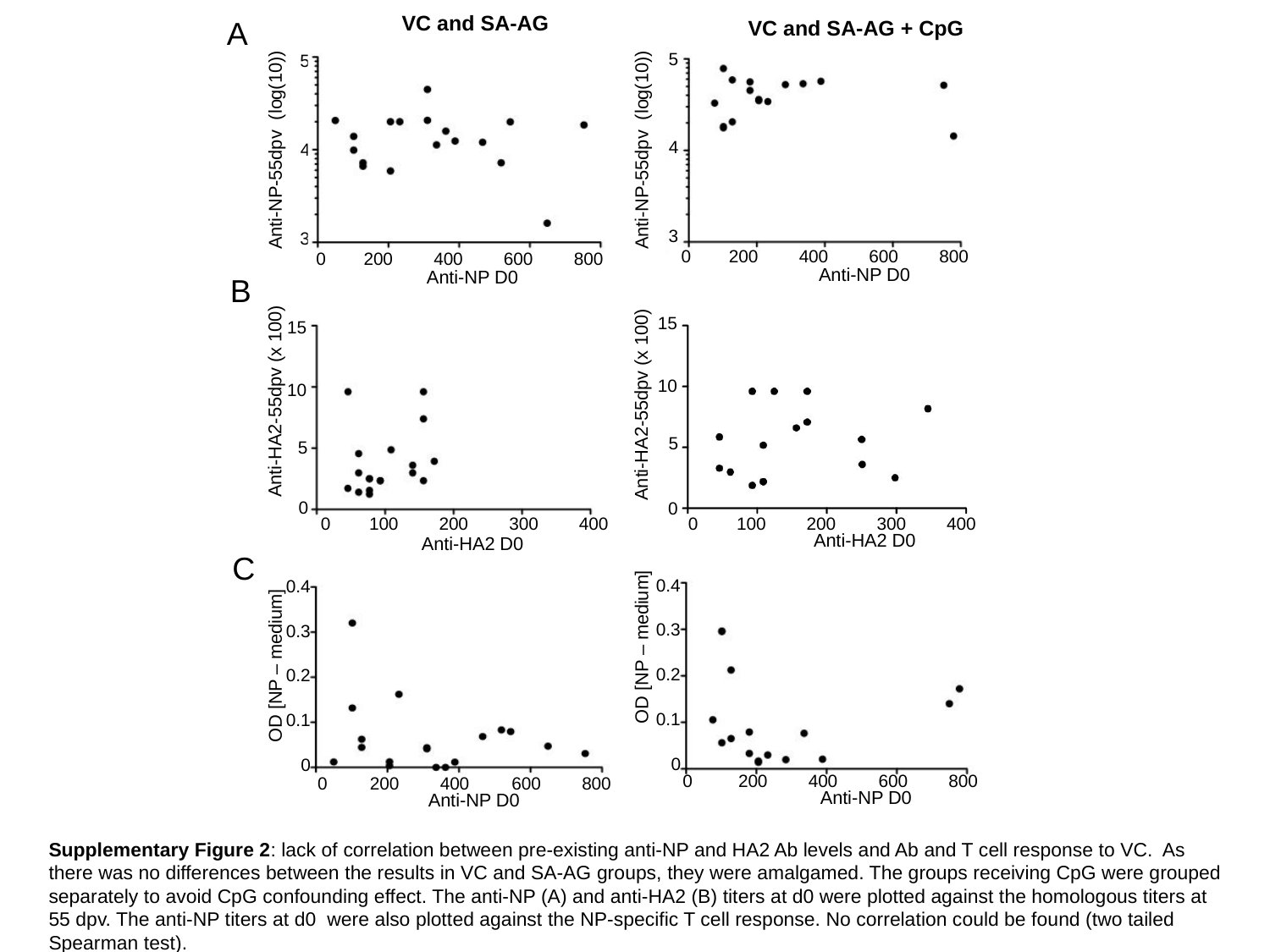

VC and SA-AG
A
VC and SA-AG + CpG
5
4
3
5
4
Anti-NP-55dpv (log(10))
Anti-NP-55dpv (log(10))
3
0
200
400
600
800
0
200
400
600
800
Anti-NP D0
Anti-NP D0
B
15
15
10
10
Anti-HA2-55dpv (x 100)
Anti-HA2-55dpv (x 100)
5
5
0
0
0.4
0.3
0.2
0.1
0
0
100
200
300
400
0
100
200
300
400
Anti-HA2 D0
Anti-HA2 D0
C
0.4
0.3
0.2
0.1
0
OD [NP – medium]
OD [NP – medium]
0
200
400
600
800
0
200
400
600
800
Anti-NP D0
Anti-NP D0
Supplementary Figure 2: lack of correlation between pre-existing anti-NP and HA2 Ab levels and Ab and T cell response to VC. As there was no differences between the results in VC and SA-AG groups, they were amalgamed. The groups receiving CpG were grouped separately to avoid CpG confounding effect. The anti-NP (A) and anti-HA2 (B) titers at d0 were plotted against the homologous titers at 55 dpv. The anti-NP titers at d0 were also plotted against the NP-specific T cell response. No correlation could be found (two tailed Spearman test).

## Slide 3
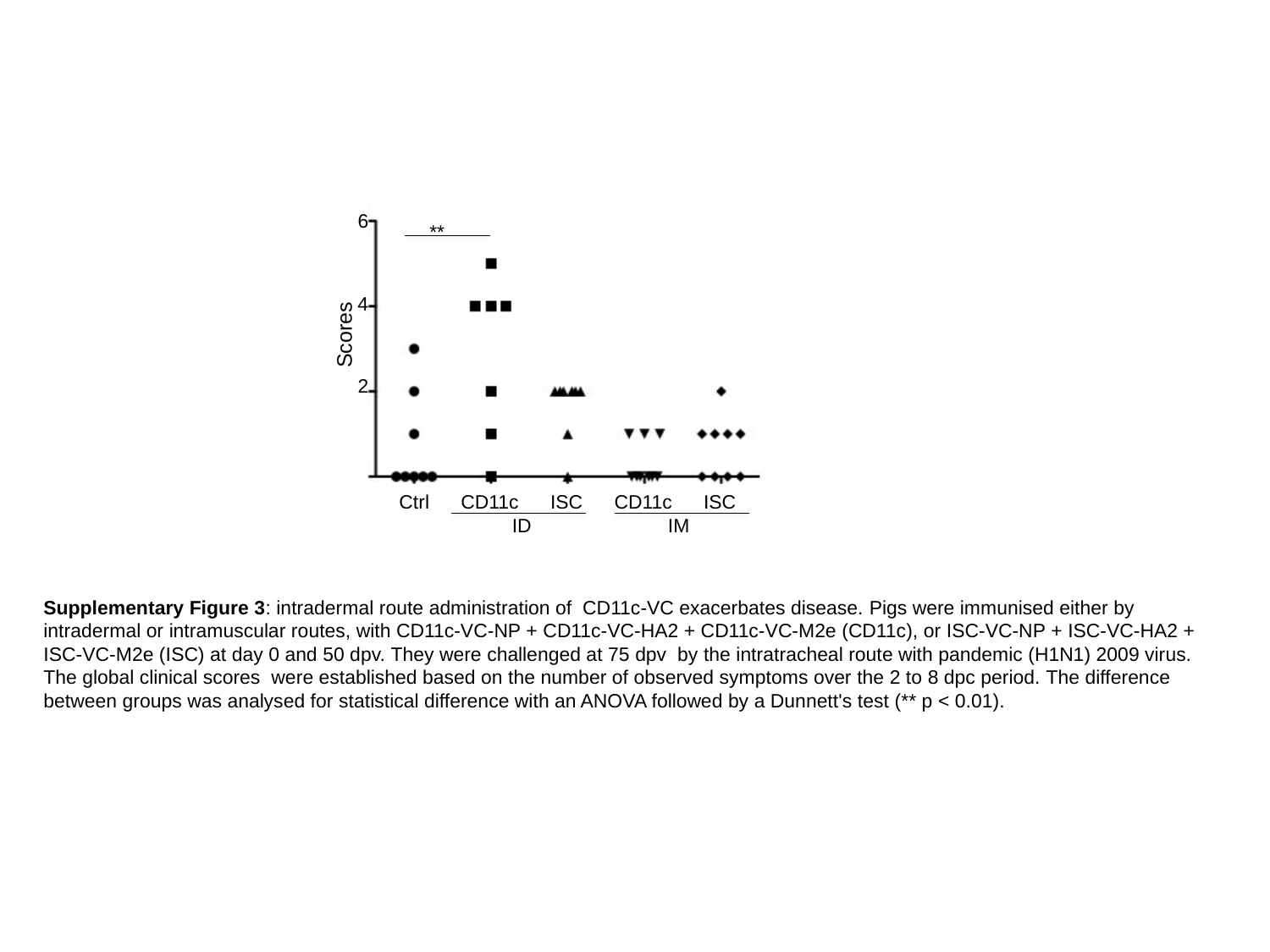

6
 **
4
Scores
2
Ctrl
CD11c
ISC
CD11c
ISC
ID
IM
Supplementary Figure 3: intradermal route administration of CD11c-VC exacerbates disease. Pigs were immunised either by intradermal or intramuscular routes, with CD11c-VC-NP + CD11c-VC-HA2 + CD11c-VC-M2e (CD11c), or ISC-VC-NP + ISC-VC-HA2 + ISC-VC-M2e (ISC) at day 0 and 50 dpv. They were challenged at 75 dpv by the intratracheal route with pandemic (H1N1) 2009 virus. The global clinical scores were established based on the number of observed symptoms over the 2 to 8 dpc period. The difference between groups was analysed for statistical difference with an ANOVA followed by a Dunnett's test (** p < 0.01).

## Slide 4
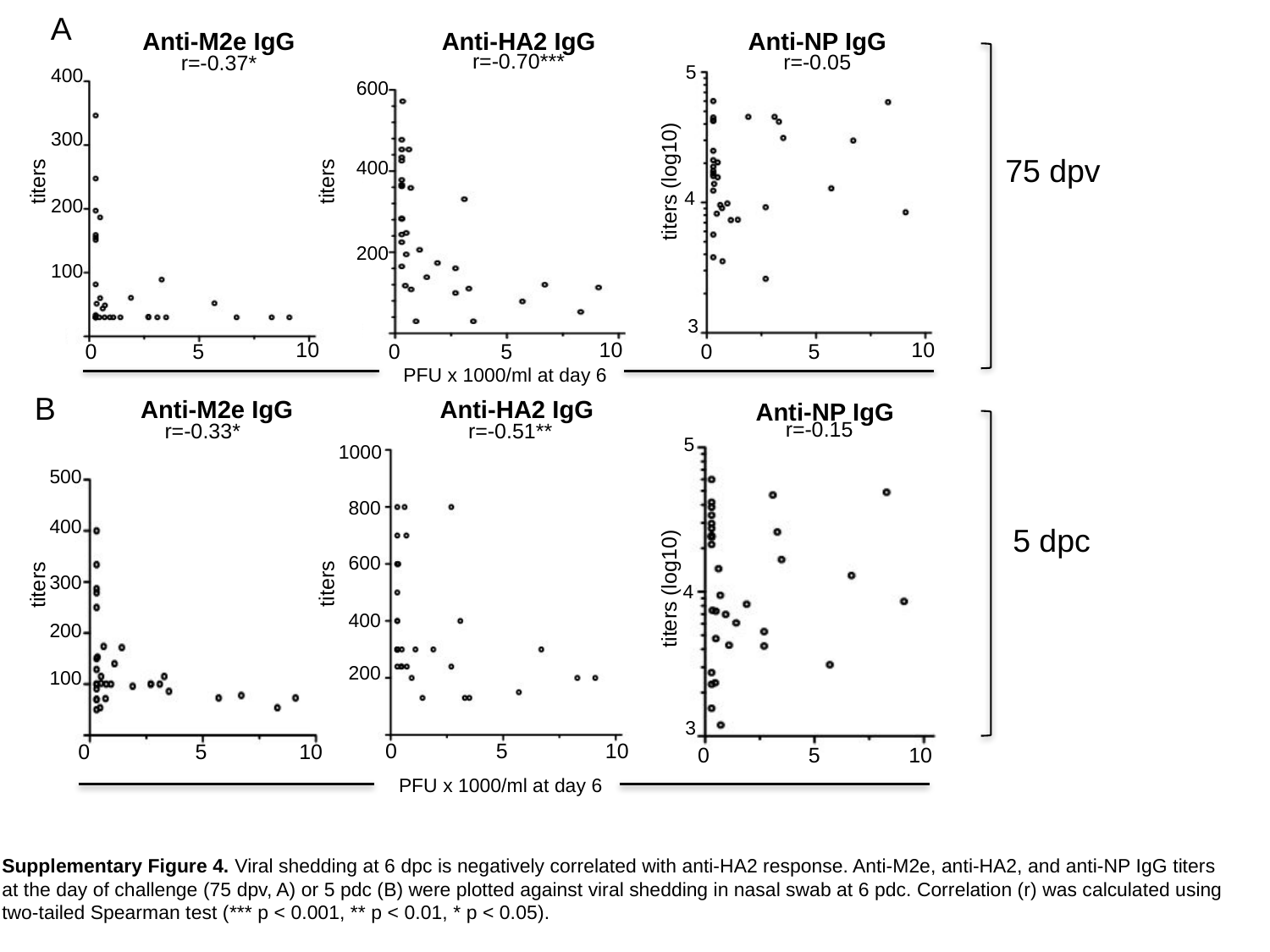

A
Anti-M2e IgG
Anti-HA2 IgG
Anti-NP IgG
r=-0.70***
r=-0.05
r=-0.37*
5
400
600
300
75 dpv
400
titers
titers
titers (log10)
4
200
200
100
3
10
0
5
10
0
5
10
0
5
PFU x 1000/ml at day 6
B
Anti-M2e IgG
Anti-HA2 IgG
Anti-NP IgG
r=-0.15
r=-0.33*
r=-0.51**
5
1000
500
800
400
300
200
100
5 dpc
600
titers
titers
titers (log10)
4
400
200
3
5
0
10
0
5
10
0
5
10
PFU x 1000/ml at day 6
Supplementary Figure 4. Viral shedding at 6 dpc is negatively correlated with anti-HA2 response. Anti-M2e, anti-HA2, and anti-NP IgG titers at the day of challenge (75 dpv, A) or 5 pdc (B) were plotted against viral shedding in nasal swab at 6 pdc. Correlation (r) was calculated using two-tailed Spearman test (*** p < 0.001, ** p < 0.01, * p < 0.05).
